# Phytochemical Analysis and Evaluation of Antioxidant and Biological Activities of Extracts from Three Clauseneae Plants in Northern Thailand

**DOI:** 10.3390/plants10010117

**Published:** 2021-01-08

**Authors:** Keerati Tanruean, Pisit Poolprasert, Nakarin Suwannarach, Jaturong Kumla, Saisamorn Lumyong

**Affiliations:** 1 Biology Program, Faculty of Science and Technology, Pibulsongkram Rajabhat University, Phitsanulok 65000, Thailand; keerati.t@psru.ac.th (K.T.); poolprasert_p@psru.ac.th (P.P.); 2 Research Center of Microbial Diversity and Sustainable Utilization, Faculty of Science, Chiang Mai University, Chiang Mai 50200, Thailand; suwan.462@gmail.com (N.S.); jaturong_yai@hotmail.com (J.K.); 3 Department of Biology, Faculty of Science, Chiang Mai University, Chiang Mai 50200, Thailand; 4 Academy of Science, The Royal Society of Thailand, Bangkok 10200, Thailand

**Keywords:** tribe Clauseneae, DNA barcode, volatile compounds, antioxidant activity, ACE inhibitory activity, anticancer activity, *α-*glucosidase inhibitory activity

## Abstract

This study established the DNA barcoding sequences (*mat*K and *rbc*L) of three plant species identified in the tribe Clauseneae, namely *Clausena excavata*, *C. harmandiana* and *Murraya koenigii*. The total phenolic and total flavonoid contents, together with the biological activities of the derived essential oils and methanol extracts, were also investigated. Herein, the success of obtaining sequences of these plant using two different barcode genes *mat*K and *rbc*L were 62.5% and 100%, respectively. Both regions were discriminated by around 700 base pairs and these had resemblance with those of the Clausenae materials earlier deposited in Genbank at a 99–100% degree of identity. Additionally, the use of *mat*K DNA sequences could positively confirm the identity as monophyletic. The highest total phenolic and total flavonoid content values (*p <* 0.05) were observed in the methanol extract of *M. koenigii* at 43.50 mg GAE/g extract and 66.13 mg QE/g extract, respectively. Furthermore, anethole was detected as the dominant compound in *C. excavata* (86.72%) and *C. harmandiana* (46.09%). Moreover, anethole (26.02%) and caryophyllene (21.15%) were identified as the major phytochemical compounds of *M. koenigii*. In terms of the biological properties, the *M. koenigii* methanol extract was found to display the greatest amount of antioxidant activity (DPPH; IC_50_ 95.54 µg/mL, ABTS value 118.12 mg GAE/g extract, FRAP value 48.15 mg GAE/g extract), and also revealed the highest *α-*glucosidase and antihypertensive inhibitory activities with percent inhibition values of 84.55 and 84.95. Notably, no adverse effects on human peripheral blood mononuclear cells were observed with regard to all of the plant extracts. Furthermore, *M. koenigii* methanol extract exhibited promise against human lung cancer cells almost at 80% after 24 h and 90% over 48 h.

## 1. Introduction

Oxidative stress and autoxidation are known to be harmful to the human body. The presence of these conditions can result from an imbalance between free radicals and antioxidants present in the body, which can ultimately be damaging to the human body’s cells, proteins and DNA. This set of circumstances can lead to a range of health problems such as aging, diabetes and cancer, as well as certain cardiovascular and neurogenerative diseases [1,2,3]. To treat these disease, synthetic compounds can be used to help prevent any effects resulting from the specific mechanisms that may occur. In spite of their advantages, it has been recently reported that an overdose of chemical compounds might lead to a range of detrimental side effects and health issues including the formation of carcinogenic tissues or tumors [4]. Consequently, natural products have been recognized for their potential in the development of medicinal treatment to address many of these illnesses. In this way, the active molecules derived from plants and other natural resources have been isolated and enhanced with the intention of reducing the oxidation processes that occur in these plant substances and to development alternative and beneficial forms of treatment. The Clauseneae tribe belongs to the Rutaceae family, which is generally recognized as a citrus family of flowering plants (Angiosperms). The tribe Clauseneae is comprised of five genera, namely *Micromelum*, *Marillia*, *Glycosmis*, *Murraya* and *Clausena* [5]. The latter genus has been identified as a potent natural resource. It has been used in a variety of applications and is considered a traditional food source. It is now being considered for use in the field of pharmacology. Furthermore, there is a great need to discover and develop novel bioactive compounds that can be utilized for a range of medicinal purposes including for anticancer, antibacterial, antifungal and anti-malarial treatments [6,7,8]. *Clausena* is a relatively small genus of strongly scented evergreen trees with odd-pinnate leaves. It is comprised of around 30 species that are distributed throughout tropical Asia, South Asia and South East Asia [6,9] and are primarily found in India. In Thailand, the genus *Clausena* is considered an economically important group of plants with a range of potential health benefits. Specifically, *C. excavata* and *C. harmandiana* have been identified as plants within this genus that show potential in the development of natural health treatments [10,11]. Concerning the tribe Clauseneae, several species in this tribe have been acknowledged as sources of edible fruits, essential oils, herbal medicines, spices (*Murraya exotica* and *M. kwangsiensis*) and various horticultural items (*Clausena anisum-olens*, *C. lansium* and *Glycosmis parviflora*). Several species have also been recognized as potential sources of lumber and pharmaceutical compounds [12]. Additionally, *Murraya koenigii*, a popular and commonly known species, is scattered throughout different regions of the globe [13]. Recently, Balakrishnan et al. [14] speculated that *M. koenigii* was a beneficial source of various bioactive compounds such as alkaloids, polyphenols, terpenoids and flavonoids. Additionally, *M. koenigii* was also found to be capable of expressing a range of anticarcinogenic, proapoptotic, antiangiogenic, antimetastatic, immunomodulatory and antioxidant properties. These properties can be further developed as antiemetic and antidiarrheal agents. They could then potentially be used to treat diseases like dysentery as a febrifuge, blood purifier, tonic and stomachic. Furthermore, *M. koenigii* is already being used as a flavoring agent in curries and chutneys. In some cultures, *M. koenigii* is being utilized in traditional systems of medicine [15].

Over the last decades, molecular technique based on DNA sequence can be applied to the systematic identification of known and unknown plants and animals. DNA barcoding, a modern method for the rapid identification of any species, is considered as a molecular and bioinformatics tool for species differentiation, identification and discovery of new species at molecular systematic level [16]. In terms of barcoding of vascular plants, it was mostly focused on markers of chloroplast genes, several markers were assessed and with time most commonly applied combinations are *rbc*L, *mat*K, *trn*H*-psb*A, with a nuclear internal transcribed spacer 2 (ITS2) established [17,18,19,20].

As mentioned above, bioactivities, namely anticarcinogenic and antioxidant have extensively addressed in the Clauseneae. Nonetheless, many aspects among these plants such as chemical profiles, some biological properties and DNA evident are still scarce and they are needed to be explored.

Nonetheless, due to a set of resembling systematics, this tribe has been labeled with a diverse range of local names at the local level, which can mislead people and result in incidences of misidentification. Furthermore, similar plant species have been utilized for numerous alternative purposes as ingredients in food or in pharmaceuticals. This present study, therefore, has aimed to confirm the obscure major taxa of Clauseneae i.e., *Clausena excavata*, *C. harmandiana* and *Murraya koenigii* gathered from Chiang Mai, Thailand using the DNA barcoding sequences obtained from two candidates of barcoding loci (*mat*K and *rbc*L). In addition, phytochemical screening and an assessment of the biological properties of these Clauseneae plants were also carried out.

## 2. Results and Discussion

### 2.1. Genetic Variation Analysis

**Figure 1:**
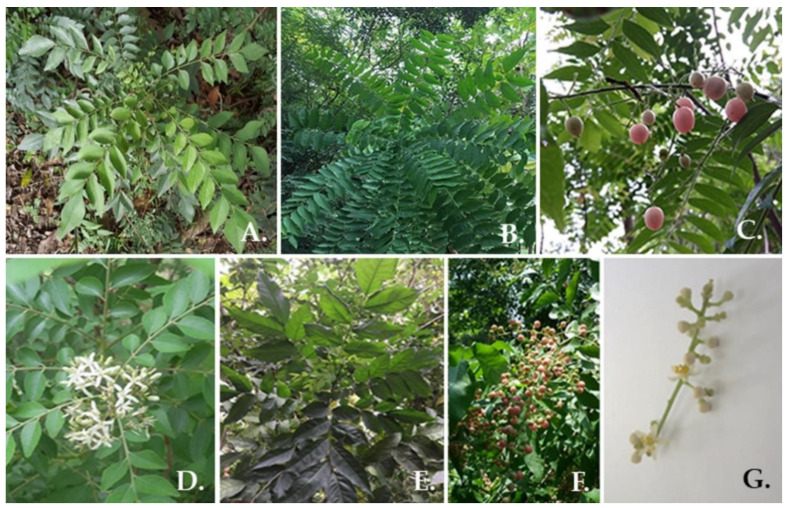
General morphology of plants. (**A**) PSRU-RUT001 (foliage of unknown plant species), (**B**), *Clausena excavata* plant (PSRU-RUT003), (**C**) Fruits of *C. excavata*, (**D**) flowers of *C. excavata*, (**E**) *Clausena harmandiana* plant (PSRU-RUT005), (**F**) Fruits of *C. harmandiana* and (**G**) flowers of *C. harmandiana.*

Botanically, three plant species in the Clauseneae tribe have been recognized as a type of strongly fragrant evergreen trees with a tall slender shape of approximately 10 m tall, and odd-pinnate narrow leaves positioned in around 10–15 pairs separated to slanting leaf-lets that are 3.5 to 7 cm long. All materials were morphologically confirmed by an expert botanist by using the Forest Trees of Southern Thailand [21]. It was determined that the specific collected materials labeled with voucher numbers PSRU-RUT001-PSRU-RUT002 could not be identified by species. However, voucher numbers PSRU-RUT003-PSRU-RUT004 and PSRU-RUT005-PSRU-RUT006 could be identified as *Clausena excavata* and *C. harmandiana*, respectively. The details of the morphological features of each representative Claseneae tree are presented in Figure 1.

**Figure 2:**
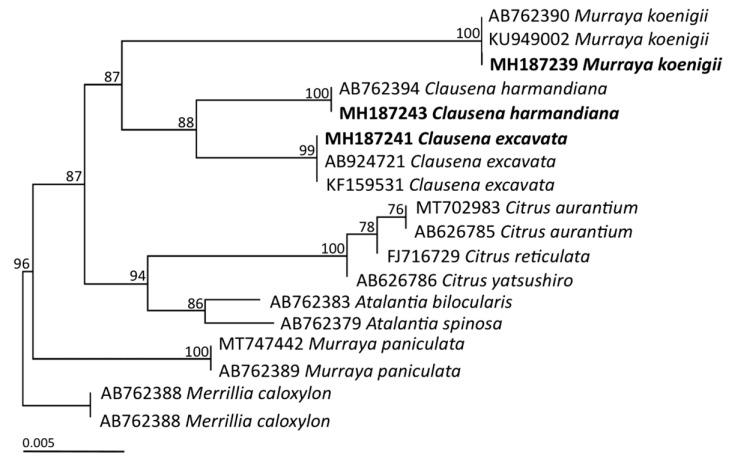
Phylogenetic tree derived from maximum likelihood analysis of the *mat*K gene of 18 sequences. Bar represents 0.005 substitutions per nucleotide position. The plants from this study are presented in bold.

Furthermore, their relevant molecular traits were assessed to identify all plant specimens. Herein, we have established the DNA barcoding sequences (*mat*K and *rbc*L) of these three species of plants to be in the tribe Clauseneae. All obtained sequences were verified by their accession numbers (*mat*K; MH187239- MH187243 and *rbc*L; MH187222- MH187229) and compared to the sequences previously deposited in Genbank using BLAST. Regarding molecular identification, genomic DNA was successfully extracted from every specimen. Two main chloroplast *mat*K and *rbc*L genes were employed for the purposes of DNA barcoding. Upon PCR amplification, fragments encompassing both *mat*K and *rbc*L were distinguished by approximately 700 base pairs. The success rates of PCR amplification of *mat*K and *rbc*L were 62.5% and 100%, respectively. Regions of similarity between the sequences were observed via the BLAST program. It was determined that these were similar to those of the *Clausena*e specimens previously deposited in Genbank at a 99–100% degree of accuracy. Based on *mat*K DNA sequences, five samples of Clauseneae (PSRU_RUT001-PSRU_RUT002, PSRU_RUT003-PSRU_RUT004 and PSRU_RUT005) were found to be closely related to *Murraya koenigii* (L.) Spreng, *Clausena excavata* Burm.f. and *Clausena harmandiana* (Pierre) Pierre ex Guillaumin, respectively. Furthermore, eight *rbc*L sequences revealed a close resemblance to *M. koenigii* (L.) Spreng (PSRU_RUT001-PSRU_RUT002), *C. excavata* Burm.f. (PSRU_RUT003-PSRU_RUT004) and *C. harmandiana* (Pierre) Pierre ex Guillaumin (PSRU_RUT005-PSRU_RUT008). Nevertheless, some *rbc*L sequences were observed to be similar to the sequences previously deposited in Genbank, namely *M. koenigii* (99.58%, complete genome), *M. paniculate* (99.58%, complete genome) and *Micromelum minutum* (99.44%, complete genome) etc. This set of circumstances resulted in a number of difficulties with regard to establishing the correct species name. Therefore, representative specimens obtained from the *mat*K DNA sequences [PSRU-RUT001 (MH187239), PSRU-RUT003 (MH187241) and PSRU-RUT005 (MH187243)] and a relevant outgroup were further assessed by phylogenic analysis using the maximum likelihood (ML) method with 1000 bootstrap replicates. The phylogenic construction revealed that each representative plant species was reciprocally monophyletic as depicted in Figure 2.

In the past decade, DNA barcoding inferred from nuclear ribosomal internal transcribed spacer (ITS) sequence was employed for the discrimination of *M. koenigii*. It was suggested that the ITS sequence was an appropriate molecular marker for isolation of *M. koenigii* from selected plants [5]. At this time, the *mat*K gene was determined to be more reliable when applied as a DNA barcode for the identification of the designated three plant species in the tribe Clauseneae. It was noted that the *rbc*L sequences were inconsistent with the basic principles of DNA barcoding because of certain drawbacks, for instance low rates of variation and amplification, poor universality of the primer, gene deletion and a potential number of other issues as has been postulated by Shivakumar et al. [17]. Although *mat*K had a higher discriminatory power than *rbc*L, it was more difficult to amplify it across distantly related species [18]. In the same manner, this result conformed to those of previous studies conducted by Penjor et al. [19] who established the phylogenetic relationships of citrus plants and their relatives inferred from *mat*K gene sequences. It was found that use of *mat*K as a marker alone could establish the curry tree as *M. koenigii*. A combination of both markers, *mat*K + *rbc*L, could help to discriminate between a maximum number of species. However, a number of researchers have used the integration of the DNA barcode to classify and identify species, while several different combinations of DNA barcodes have been effectively put forward for different plants [20]. To attain a maximum discrimination rate among closely related species, a combination of Internal Transcribed Spacers (ITS + *mat*K + *rbc*L) has been suggested by Li et al. [22]. 

### 2.2. Phytochemical Analysis of Essential Oils

**Figure 3:**
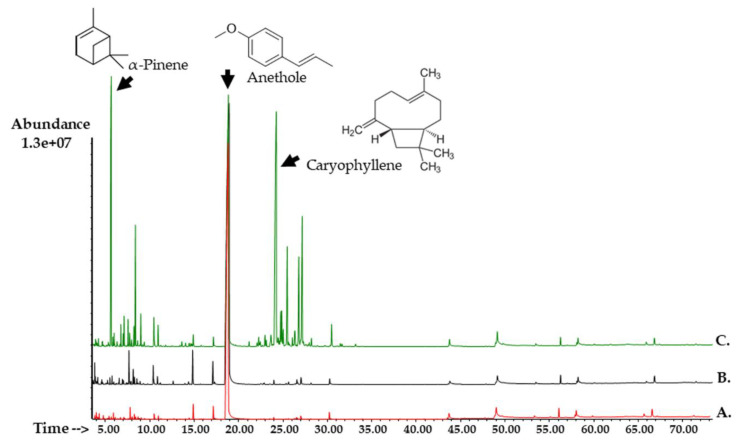
GC-MS Chromatogram of (**A**) *Clausena excavata* leaves in essential oil (**B**) *Clausena harmandiana* leaves in essential oil and (**C**) *Murraya koenigii* leaves in essential oil.

The phytochemical composition of the leaves used to obtain the essential oil of *C. excavata*, *C. harmandiana* and *M. koenigii* are shown in Table 1. A GC-MS chromatogram is presented in Figure 3. Various compounds were detected by gas chromatography. Notably, *C. harmandiana* was acknowledged as a high-performance specimen containing compounds amounting to 93.60% followed by *C. excavata* 89.86% and *M. koenigii* 88.87%. Furthermore, anethole was the most dominant compound identified in essential oil, while *C. excavata* was found to contain the highest yield at 86.72% followed by *C. harmandiana* and *M. koenigii* at 46.09% and 26.02%, respectively.

As determined by the results, anethole was identified as a major phytochemical compound of *C. excavata* and *C. harmandiana*. Additionally, minor compounds were observed to be present, namely Camphene (9.61%), *β-*Terpinene (7.87%), D-Limonene (7.07%), *β-*Mycene (3.69%), *γ-*Terpinene (3.57%), 4-Terpinenol (3.34%) and others, of which less than 3% were found in the essential oils. Cheng et al. [23] reported that the essential oil obtained from the fresh leaves of *C. excavata* contained Safrole (75.85%) and Terpinolene (17.86%) as the primary components, while Trung et al. [24] discovered that *β-*Caryophyllene (16.7%), Spathulenol (11.9%) and Bicyclogermacrene (7.5%) were the major constituents of the *C. excavata* plant collected in Vietnam. On the other hand, Arbab et al. [6] revealed that *α-*Pinene (12.23%) and Copaene (12.40%) were the major constituents present in the essential oil of *C. harmandiana*. Moreover, bioactive Carbazoles and Coumarins were observed as the major constituents of *C. excavata* and *C. harmandiana*, of which these two compounds were identified as bioactive compounds [11,25,26]. According to the results of the assessment of the essential oil of *M. koenigii*, it was found to possess Anethole (26.02%), Caryophyllene (21.15%) and *α-*Pinene (12.23%) as the major components. These results are in accordance with those presented in the published report of Sukkaew et al. [27], wherein the essential oil obtained from the fresh leaves of *M. koenigii* collected from Surat Thani Province, Thailand, was found to contain *β-*Caryophyllene (21.4%), *α-*Selinene (10.2%) and *α-*Humulene (7.1%) and *α-*Pinene (4.4%) as its major components. However, in this study, the chemical composition of the essential oil of *M. koenigii* was distinctly different from that of previous studies. The composition of the essential oils of the fresh leaves of *M. koenigii* that was cultivated at six locations in Peninsula Malaysia and Borneo revealed that the two major volatile metabolites were identified as *β-*Caryophyllene (16.6–26.6%) and *α-*Humulene (15.2–26.7%). The volatile substances could be categorized as sesquiterpene hydrocarbons and oxygenated sesquiterpenes as the major groups along with oxygenated monoterpenes and oxygenated diterpenes [28], Caryophyllene (9.5%), *β-*Myrcene (3.2%), *α-*Caryophyllene (2.8%), 4-Terpineol (2.8%), *g*-Terpinene (2.7%) and Allyl (methoxy) dimethylsilane (2.6%) [29]. Rao et al. [13] stated that the essential oil obtained from wild and cultivated *M. koenigii* leaves collected from ten Indian locations revealed different chemical compositions. For the essential oil of the wild plants, *α-*Pinene (55.7%) and *β-*Pinene (10.6%) were detected as the dominant constituents.

Furthermore, *α-*Pinene (13.5–35.7%) and/or *β-*Phellandrene (14.7–50.2%) were identified as the major components of the specimen collected from seven other locations. Additionally, (E)-Caryophyllene (26.5%–31.5%) and *α-*Selinene (9.5%–10.4%) were considered the principal components of the essential oils derived from the specimens collected from two locations. The chemical compositions of the essential oil of two Chemotypes of *M. koenigii* collected from northern India were assessed in different seasons. Chemotype A contained the major compounds of *α-*Pinene (34.6–41.9%), Sab-inene (26.1–36.1%), (E)-Caryophyllene (2.4–5.4%) and Terpinen-4-ol (1.5–5.3%), while chemotype B contained *α-*Pinene (52.7–65.3%), *β-*Pinene (10.7–12.9%), (E)-Caryophyllene (3.1–10.3%) and Limonene (5.1–5.7%) [30]. On the other hand, the major compounds detected in the essential oil of *M. koenigii* leaves collected from southern India, namely Linalool (32.83%), Elemol (7.44%), Geranyl acetate (6.18%), Myrcene (6.12%), Allo-Ocimene (5.02%), *α-*Terpinene (4.9%), (E)-*β-*Ocimene (3.68%) and Neryl acetate (3.45%), were also identified [31]. Notably, Tripathi et al. [32] established that 3-Carene (18.52%), *β-*Pinene (13.57%), *α-*Pinene (9.38%), Linalool (5.42%), *α-*Eudesmol (4.55%), ρ-Cymene (3.61%), *γ-*Terpinene (3.48%), *α-*Amorphene (3.38%), Allo-Ocimene (2.75%), Sabinene (2.55%), *γ-*Terpinene (2.48%), Linalyl acetate (2.46%), Myrcene (2.43%) and *β-*Eudesmol (2.16%) were the crucial constituents of the essential oil of *M. koenigii* collected from Uttarakhand, India. Hence, variations in the compositions of the essential oils of curry leaves were found to be dependent upon season and the specific location of collection.

### 2.3. Total Phenolic and Total Flavonoid Contents

The total phenolic (TPC) and total flavonoid contents (TFC) in the extracts of the leaves of *C. excavata*, *C. harmandiana* and *M. koenigii* were expressed as mg gallic acid equivalent (GAE) and mg quercetin equivalent (QE) per gram extract. As is shown in Table 2, TPC ranged from 7.07 to 43.50 mg GAE/g extract, of which *M. koenigii* in the methanol extract exhibited the highest yields amounting to 43.50 mg GAE/g extract (*p* < 0.05) followed by *M. koenigii* in essential oil, *C. excavata* in methanol extract, *C. har-mandiana* in methanol extract, *C. excavata* in essential oil and *C. harmandiana* in essen-tial oil (32.28, 22.89, 19.71, 9.70 and 7.07 mg GAE/g extract, respectively). Meanwhile, TFC ranged from 16.82 to 66.13 mg QE/g extract *M. koenigii* in the methanol extract and presented the highest value at 66.13 mg QE/g extract (*p* < 0.05) followed by *M. koenigii* in essential oil, *C. harmandiana* in methanol extract, *C. excavata* in methanol extract, *C. excavata* in essential oil, and *C. harmandiana* in essential oil (50.57, 39.95, 30.89, 23.91 and 16.82 mg QE/g extract, respectively). Additionally, the phenolic compounds and flavonoids that were found in the three Clauseneae extracts, along with certain other phytoconstituents, such as phenols, steroids, saponins, quinones, alkaloids, flavonoids, tannins, carbohydrates, proteins and volatile oils, were found to be present in the Clauseneae plants [33].

Antioxidant activity of the essential oils and methanol extracts of three Clauseneae plants were measured by DPPH, ABTS and FRAP assays. In terms of establishing potential antioxidant activities, the DPPH free radical scavenging ability is usually employed as the preferred method. In this situation, the antioxidant compounds in the plant extracts donate an electron or hydrogen radical to an unstable DPPH free radical and then become a stable diamagnetic molecule [34]. In this study, the DPPH free radical scavenging activity of the three Clauseneae plant extracts were expressed as IC_50_ (µg/mL). The DPPH radical scavenging activities of *C. excavata*, *C. harmandiana* and *M. koenigii* in the methanol extracts and essential oils were observed and are presented in Table 3. Specifically, the IC_50_ values ranged from 95.54 µg/mL to 2865.26 µg/mL. Furthermore, it was revealed that *M. koenigii* in the methanol extract displayed the highest radical scavenging activity at 95.54 µg/mL followed by *M. koenigii* in essential oil (167.74 µg/mL), *C. excavata* in methanol extract (904.53 µg/mL), *C. harmandiana* in methanol extract (2037.66 µg/mL), *C. excavata* in essential oil (2059.29 µg/mL) and *C. harmandiana* in essential oil (2865.26 µg/mL). There was a strong correlation between the DPPH free radical scavenging activity in terms of the total phenolic (r = 0.810, *p* < 0.05) and total flavonoid (r = 0.835, *p* < 0.05) contents of all plant extracts. Furthermore, the antioxidant capacities of the essential oil and methanol extracts of the three Clauseneae plants were investigated through the scavenging activity of the ABTS cation free radicals. With regard to the mechanism of this assay, the antioxidant compounds donated an electron and hydrogen atom to an unstable ABTS^+^ cation radical, which could then convert to a stable ABTS radical form and then be used to evaluate the degree of antioxidant activity by reduction of the bluegreen ABTS^+^ radical. The ABTS free radical scavenging ability of various extracts of the Clauseneae plant extracts was expressed as mg GAE/g extract. The ABTS cation scavenging activity of the methanol extract of *M. koenigii* was found to be significantly higher than the other extracts (*p* < 0.05) at a value of 118.12 mg GAE/g extract, followed by the methanol extracts of *C. excavata* (88.65 mg GAE/g extract) and *C. harmandiana* (80.11 mg GAE/g extract).

Furthermore, the essential oil of all plants displayed less scavenging activity of the ABTS cation radical than the methanol extracts, with the exception of the essential oil extract of *M. koenigii* which possessed a degree of high ABTS cation radical that was close to that of the methanol extract of *C. harmandiana* (*p* > 0.05). Importantly, a high correlation between ABTS cation free radical scavenging activity with total phenolic (r = 0.881, *p* < 0.05) and total flavonoid (r = 0.880, *p* < 0.05) contents of all plant extracts was observed. Additionally, the antioxidant activity of Clauseneae plant extracts was investigated by ferric reducing antioxidant power (FRAP) assay based on the measurement of the ability of antioxidants to reduce ferric iron (Fe^3+^) to its ferrous (Fe^2+^) form. The potential antioxidant activity was then measured through its reducing ca-pacity. The FRAP values of the extracts were expressed as mg GAE/g extract. The highest FRAP value was found in the methanol extract of *M. koenigii* (48.15 mg GAE/g extract) (*p* < 0.05), while the essential oil of *C. excavata* displayed a minimum degree of antioxidant activity at 5.07 mg GAE/g extract. Additionally, a high correlation was observed between their FRAP values and their total phenolic (r = 0.823, *p* < 0.05) and total flavonoid (r = 0.875, *p* < 0.05) contents. It was further observed that the methanol extract of *M. koenigii* showed strong DPPH free radical scavenging activity, the highest degree of ABTS free radical scavenging ability, and also exhibited high reducing power. All of which indicated that the methanol extract of *M. koenigii* has the potential to scavenge both neutral and cation free radicals. Moreover, a significant correlation between chemical constituents and antioxidant activity suggests that the phenolic and flavonoid contents of Clauseneae plants extracts are associated with their strong antioxidant activities. In addition, the antioxidant activities of the extracts may be a result of the level of terpene contained in the extract. A previous study conducted by Ma et al. [35] revealed that the alkenes isolated from *M. koenigii* exhibited significant antioxidant activities using DPPH free radical scavenging assay. The study conducted by Truong et al. [36] reported that the methanol extract of *Severinia buxifolia*, belonging to the Rutaceae family, showed the highest extraction yield along with the highest content of phenolics, flavonoids, alkaloids and terpenoids. Moreover, the methanol extract also possessed the highest degree of antioxidant activity indexed by the DPPH free radical scavenging assay and the highest in vitro anti-inflammatory activity. Similarly, Albaayit et al. [37] speculated that the extract of the methanol leaves of *C. excavata* displayed the highest phenolic content and antioxidant activity based on FRAP and DPPH radical scavenging activity assays. These finding support the contention that methanol is the optimal solvent for the extraction of Rutaceae plants.

The *α-*glucosidase inhibitory activities of *C. excavata*, *C. harmandiana* and *M. koenigii* in the methanol extract and essential oil are shown in Table 4. The results reveal that the percentage of inhibition of *α-*glucosidase inhibitory activities ranged from 24.33% to 84.55% and the methanol extract of *M. koenigii* displayed positive inhibitory activity at 84.55% inhibition. The antidiabetic activity of Claseneae plants has been reported, while the root ethanolic extract of *C. excavata* that contained dentatin and heptaphyllin exhibited high activity in term of *α-*glucosidase inhibitory [38]. Specifically, the extract of *M. koenigii* exhibited high potential in preventing diabetes [39,40].

Furthermore, the inhibition of *α-*glucosidase, an important enzyme that hydrolyses oligosaccharides, can reduce the risk of diabetes and obesity. Based on our results, the high potential of *α-*glucosidase inhibitory activity observed in the methanol extract of *M. koenigii* could be related to its high total phenolic and total flavonoid contents. This would be indicative of strong relative correlations of 0.792 and 0.804 (*p* < 0.05). These results are supported by the findings of Nagarani et al. [41], which suggested that the *α-*glucosidase inhibitory activity of natural substances was strongly correlated with the contents of the flavonoid and phenolic compounds. Moreover, antihypertensive inhibitory activity was investigated and the percentage ranged from 31.07% to 84.95% (Table 4). The methanol extract of *M. koenigii* recorded the highest value at 84.95%. These results revealed a strong correlation r = 0.704 (*p* < 0.05) and r = 0.727 (*p* < 0.05) between the antihypertensive inhibitory activity of all plant extracts and their total phenolic and total flavonoid contents, respectively. Moreover, the high potential of the antihypertensive (>80%) properties of plants has been reported in various plant species such as *Quercus infectoria* (Fagaceae) (93.9%), *Berberis integerrima* (Berberidaceae) (88.2%), *Crataegus microphylla* (Rosaceae) (80.9%) and *Onopordon acanthium* (Asteraceae) (80.2%) [42].

### 2.4. Determination of Methanol Extract and Essential Oil of Clausena excavata, Clausena harmandiana and Murraya koenigii against Human Normal Cells and Human Lung Cancer Cells

Over the past decade, the number of cancer deaths worldwide has been increasing. It is a significant cause of many of the health problems experienced by humans around the world. Cancer is followed by a range of cardiovascular diseases in terms of mortality rates. Notably, the leading cause of deaths attributed to cancer is lung cancer. Therefore, the antitumor activity of the three Clauseneae plant extracts in this study were investigated. The effect of the essential oil and methanol extracts of *C. excavata*, *C. harmandiana* and *M. koenigii* by varied concentrations (0–1 µg/mL) were observed for 24 and 48 h against human normal cells using human peripheral blood mononuclear cell (PBMC) and human lung cancer cell (A549).

**Figure 4:**
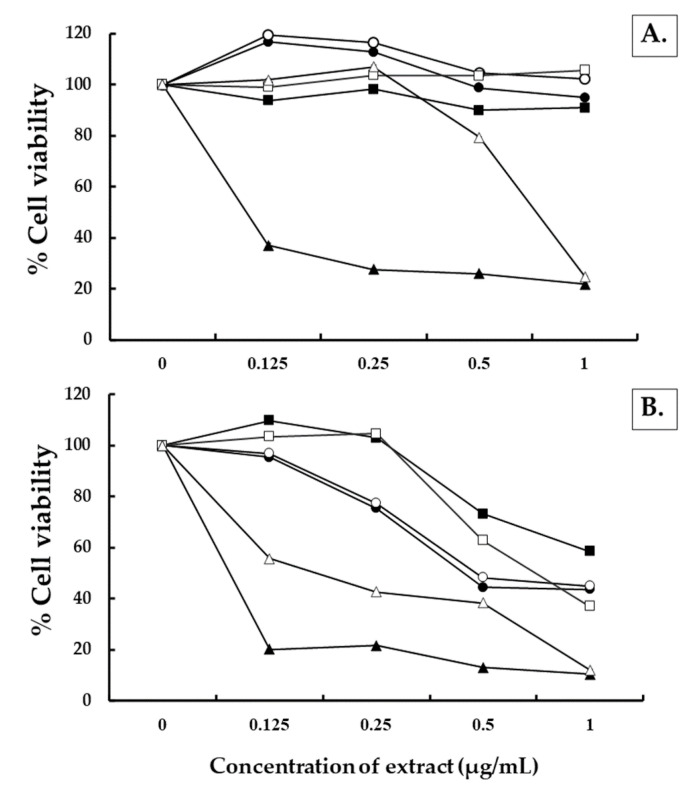
Effects of the extracts of *Clausena excavata* (essential oil, -○-; methanol extract, -●-), *Clausena harmandiana* (essential oil, -□-; methanol extract, -■-) and *Murraya koenigii* (essential oil, -△-; methanol extract, -▲-) against human lung cancer cells (A549) at 24 h (**A**) and 48 h (**B**).

The results revealed that the essential oil and methanol extracts of *C. excavata*, *C. harmandiana* and *M. koenigii* did not have any toxic effect on PBMC at a concentration of 1 µg/mL relating to the increasing number of cell viability after being tested over 24 h and 48 h (data not shown). For A549 cells assessment, the essential oil and methanol extracts of *C. excavata*, *C. harmandiana* and *M. koenigii* could effectively decrease cell viability when concentrations were increased to 1 µg/mL (Figure 4). This was especially true for *M. koenigii* in the methanol extract, which displayed high efficacy activity against A549 cells at almost 80% after 24 h and 90% over 48 h. The study conducted by Huang et al. [43] determined that carbazole alkaloids and coumarins obtained from *Clausena* plants exhibited anticancer activity. Based on the finding of our study, all the Clauseneae plant extracts exhibited high activity against human lung cancer cells. This was especially true of the methanol extract of *M. koenigii* that displayed high potential activity against A549 cells when compared with the methanol leaves and stem extracts of Marsdenia glabra Cost. In turn, this extract displayed low inhibitory activity within a range of about 15–25% at 1000 µg/mL [44]. Moreover, the study conducted by Muthumani et al. [45] revealed that the extract of *M. koenigii* displayed a high degree of potential anticancer activity in experimental animal trials.

## 3. Materials and Methods

### 3.1. Plant Materials and Chemicals

All plant samples were tentatively identified to species level by comparison with the reference materials at the herbarium of Queen Sirikit Botanic Garden, Chiang Mai, Thailand, and deposited as voucher specimens (PSRU-RUT001-008) in the collection of Faculty of Science and Technology, Pibulsongkram Rajabhat University (PSRU), Phitsanulok Province, Thailand.

Gallic acid was purchased from Merck (Germany) and folin-ciocalteu reagent was bought from BDH Chemicals Ltd. (Poole, England). The 2,2′-Diphenyl-1-picrylhydrazyl radical (DPPH) and 2,4,6-tris(2-pyridyl)-s-triazine (TPTZ) were purchased from Fluka (Steinheim, Germany). 2, 2′-Azino-bis (3-ethylbenzothiazoline-6-sulfonic acid) (ABTS) was derived from SIGMA (Oakville, ON, Canada). Intestinal acetone powder and 3-(4,5-dimethylthiazol-2-yl)-2,5-diphenyltetrazolium bromide were purchased from Sigma-Aldrich Chemical Co. (St. Louis, MO, USA). Dulbecco’s modified Eagle medium and fetal bovine serum were purchased from Invitrogen Corp. (Grand Island, NY, USA). All the solvents and other chemicals were an analytical grade (A.R.).

### 3.2. Collection of Plant Material for Genetic Variation Analysis

#### 3.2.1. Plant Sample Collection

**Figure 5:**
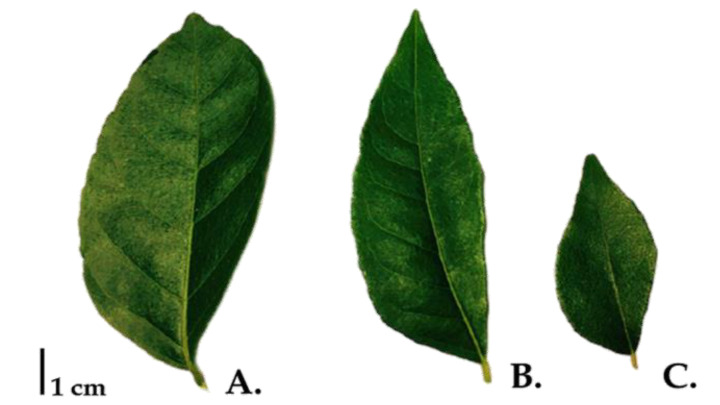
Representative leaf images of three plant species in Claseneae; (**A**) PSRU_RUT005-006, (**B**) PSRU_RUT003-004 and (**C**) PSRU_RUT001-002.

The fresh leaves were individually collected from local area in Chiang Mai province, upper northern Thailand, labeled and transported in sterile plastic bags and frozen until processed for DNA extraction. The morphological details of each representative Clauseneae leaf are demonstrated as Figure 5.

#### 3.2.2. DNA Extraction

Genomic DNA was isolated using the DNeasy Plant Mini Kit (QIAGEN, Germany) following the manufacturers protocol. Purity and quantity of the extracted genomic DNA was performed with a NanoDrop spectrophotometer. DNA was checked using 1.0% an agarose gel stained with stained with Redsafe^TM^.

#### 3.2.3. PCR Amplication

We amplified and sequenced the chloroplast DNA regions (*mat*K and *rbc*L) based on primers 1) *mat*K (*mat*K-F; 5′-TAA TTT ACG ATC AAT TCA TTC-3′ and *mat*K-R; 5′-TCT GGA GTC TTT CTT GAG CG-3′) and 2) *rbc*L (*rbc*L-F; 5′-TCA CCA CAA ACA GAA ACT AAA GC-3′ and *rbcL*-R; 5′-GGC ACA AAA TAA GAA ACG ATC TC-3′). PCR was conducted in a final reaction volume of 20 μL made up of 5× PCR Enhancer, 2 μL of 10× HF Reaction Buffer, 0.4 μL of 10 mM dNTP Mix, 0.3 μL each of the forward and reverse primers (10 μmol/L), 0.3 μL of Long and High-Fidelity DNA Polymerase (0.75 U) (biotechrabbit, Germany), 10.7 μL of nuclear free water and at least 20 ng of the DNA template. PCR amplification was performed using a T100^TM^ Thermal Cycler (BioRad, USA). The thermal profile used was: 94 °C for 3 min, 30 cycles of 94 °C for 1 min, 48 °C for 1 min, 72 °C for 1 min, and 72 °C for 10 min, and a final extension at 72 °C for 5 min. PCR products were verified on 1% agarose gels stained with Redsafe^TM^ under UV light and were then purified using GenUP PCR/Gel Cleanup Kit (Biotechrabbit, Germany). Afterwards, the purified product was direct sequenced with forward and reverse primers by Macrogen, Inc (http://www.macrogen.com).

#### 3.2.4. Alignment of Sequences and Phylogenetic Analysis

A similarity search for each sequence was verified using BLAST (https://www.ncbi.nlm.nih.gov/). The maximum likelihood (ML) methods from the MEGA (version 5.0) program [46] were used to create phylogenetic trees. The reliability of each branch was tested by bootstrap analysis with 1000 replications. Clade with bootstrap values of 70% was considered significantly supported [47]. The sequences of *Merrillia caloxyon* were used as an outgroup. All DNA sequences were finally trimmed to 718 (*mat*K) and 713 (*rbc*L) base pairs. The sequences obtained after removing the primers used for PCR amplification were deposited to NCBI-Genbank BankIt (https://www.ncbi.nlm.nih.gov/BankIt/) under accession numbers (*mat*K; MH187239-MH187243 and *rbc*L; MH187222-MH187229).

### 3.3. Preparation of Plant Extracts

The leaves of three plants of Clauseneae were dried at 50 °C for 72 h, grounded into small pieces and stored at room temperature. Plant materials (100 g) were individually extracted with 1000 mL of methanol (1:10) and left for 12 h at ambient temperature. The extracts were sonicated using an ultrasonicator (Crest, USA) for 30 min, filtered through Whatman no. 1 filter paper and evaporated under a vacuum at 40 °C using a rotary evaporator until dry. Dry extracts were kept at 4 °C in the dark for further study.

The essential oil was extracted from the dried leaves and stems by stream distillation with a ratio of 1:20 and partitioned with dichloromethane for 3 h. The essential oil was obtained after evaporation of the dichloromethane. After that, the essential oil was dehydrated with anhydrous sodium sulphate and stored at 4 °C for further analysis.

The methanol extract and essential oil of each plant species were performed in triplicate.

### 3.4. Phytochemical Analysis of Essential Oils

Phytochemical analysis of the essential oils was determined according to the previous research of Tanruean et al. [48]. The extracts were analyzed for their phytochemicals using a gas chromatography (GC) 6890 Agilent Technologies/MSD 5973 Hewlett Packard, equipped with a MS detector and an HP-5MS capillary column (bonded and cross-linked 5% phenyl-methylpolysiloxane 30 × 0.25 µm, film thickness 0.25 µm). The injector and detector temperatures were set at 270 and 280 °C, respectively. The oven temperature was set at 80 °C and held for 2 min, and then increased at a rate 10 °C/min to 120 °C and held for 4 min. The oven temperature was then in-creased at a rate 10 °C/min to 155 °C and held for 4 min, and then increased at a rate 5 °C/min to 280 °C and held for 12.50 min. The total running time was 55 min. Helium was used as a carrier gas at a flow rate of 1 mL/min. The sample (1 µL) was injected in the splitless mode. GC-MS detection of an electron ionization system with an ionization energy measurement of 70 eV was used. Injector and MS transfer line temperatures were set at 270 and 290 °C, respectively. The components were identified based on a comparison of their relative retention times and the mass spectra with W8N08 and Wiley7n libraries data of the GC-MS system.

### 3.5. Determination of Total Flavonoid Contents

The total flavonoid contents were determined by the method of Kaewnarin et al. [49] with a few slight modifications. The extract (0.5 mL) was mixed with 2 mL of methanol, followed by the addition 0.15 mL of 50 g/L NaNO_2_. After 5 min, 0.15 mL of 100 g/L AlCl_3_ was added. The reaction was mixed and incubated at ambient temperature for 15 min, and the absorbance was measured at 415 nm. Quercetin solution was used as a standard for the determination [50,51,52] and the results were expressed as mg QE/g extract. The data were presented as the average of the triplicate analyses.

### 3.6. Determination of Total Phenolic Contents

Total phenolic contents were estimated using the protocol of Thitilertdecha et al. [53] with slight modifications. The procedure involved of combining 0.25 mL of sample (1 mg/mL) with 2.5 mL of deionized water and 0.5 mL of folin-ciocalteu reagent. After 5 min, 0.5 mL of 20% (*w/v*) Na_2_CO_3_ was added, and the solution was incubated for 1 h at ambient temperature. Absorbance was then measured at 760 nm. Gallic acid solution was used as a standard for the determination and the results were expressed as mg GAE/g extract. The data were presented as the average of the triplicate analyses.

### 3.7. Determination of Antioxidant Activities

#### 3.7.1. DPPH Free Radical Scavenging Assay

The free radical scavenging ability was determined according to the method of Gülçin et al. [54] with slight modifications. The 2,2′-diphenyl-1-picrylhydrazyl radical (DPPH^●^) solution in ethanol (0.1 mM, 1.5 mL) was mixed with 0.5 mL of different concentrations of each extract, and methanol was used as the control. The mixtures were well shaken and kept at ambient temperature for 30 min in the dark. The absorbance was measured at 517 nm and gallic acid was used as the comparative standard. The percent of DPPH^●^ discoloration of the samples was calculated according to the formula:

Percent inhibition = (A_o_- A_s_/A_o_) × 100
Where A_o_ is the absorbance of the control (containing all reagents except the test compound), and A_s_ is the absorbance of the mixture containing the test compound. The test sample concentrations providing 50% inhibition (IC_50_) were calculated from the plot of inhibition percentage against extract concentration values. The radical scavenging ability was presented IC_50_ values. The data were presented as the average of the triplicate analyses.

#### 3.7.2. ABTS Radical Scavenging Activity

The ABTS^•+^ scavenging activity was measured according to the method of Re et al. [55] with some modification. The stock solution of ABTS cation chromophore was prepared by a reaction between a 7 mM ABTS solution (100 mL) and 2.45 mM potassium persulphate (final concentration) (100 mL) and was kept in a dark place at an ambient temperature for 16 h. The ABTS^•+^ solution was diluted with phosphate buffer (50 mM, pH 7.4) to an absorbance of 0.70 ± 002 at 734 nm. An aliquot of each extract (100 µL) was added to 3 mL of an ABTS^•+^ solution, and the resulting mixture was then incubated for 30 min at an ambient temperature prior to measuring the absorbance at 734 nm. Gallic acid was used as a reference compound, and the ABTS^•+^ scavenging activity was expressed as mg GAE/g extract. The data were presented as the average of the triplicate analyses.

#### 3.7.3. Ferric Reducing Antioxidant Power (FRAP) Assay

The FRAP assay was determined according to the protocol of Li et al. [56] with some modifications. The FRAP reagent containing 10 mM of 2,4,6-tris(2-pyridyl)-s-triazine (TPTZ) solution in 40 mM hydrochloric acid (20 mL), 20 mM ferric (III) chloride (20 mL) and acetate buffer (5 mL, 300 mM, pH 3.6) was pre-pared freshly prior to being used. Different concentrations of each extract (0.1 mL) was mixed with the FRAP reagent (1.5 mL) and 1.4 mL of acetate buffer (300 mM, pH 3.6) and were then incubated at an ambient temperature for 30 min. The absorbance was measured at 593 nm. Gallic acid was used as a standard and FRAP value was calculated as mg GAE/g extract. The data were presented as the average of the triplicate analyses.

### 3.8. Determination of α-Glucosidase Inhibitory Activity

*α-*Glucosidase (AGH) solution was prepared from rat intestinal acetone powder by partial modification of the procedure reported by Oki et al. [57]. 100 mg of intestinal acetone powder (Sigma-Aldrich Chemical Co.; St. Louis, MO, USA) was added to 3 mL of 0.9% NaCl solution, homogenized with the sonication and kept in an ice bath. After centrifugation at 6000 rpm for 30 min at 4 °C, the resulting supernatant was kept cold and directly subjected to inhibitory assay. The method of Adisakwattana et al. [58] was used to determine AGH inhibitory assay. The assay was defined as the percent inhibition under the assay conditions, which was calculated according to the formula:

Percent inhibition = (A_o_ − A_s_/A_o_) × 100
Where A_o_ is the absorbance of the control, and A_s_ is the absorbance of the mixture containing the test compound. The data were presented as the average of the triplicate analyses.

### 3.9. Determination of Antihypertensive Activity

The angiotensin-I-converting enzyme (ACE) inhibitory activity was evaluated by the modified method of Park et al. [59]. The sample (50 µL) was mixed with 50 µL of 25 mU/mL ACE (Sigma-Aldrich Chemical Co.; St. Louis, MO, USA) and pre-incubated at 37 °C for 10 min. Then, 6 mM hippuryl-histidyl-leucine (HHL) in 50 mM Tris with 300 mM NaCl 100 µL was added and further incubated for 30 min. The reaction was stopped by adding 200 µL of 1.0 M HCl. Hippuric acid was extracted by ethyl acetate (600 µL), followed by centrifugation at 4880 rpm for 15 min. The supernatant (200 µL) was transferred to a test tube and evaporated at 95 °C to remove the ethyl acetate. Hot distilled water (1.0 mL) was added to dissolve the hippuric acid and the absorbance was determined at 228 nm. The ACE inhibition was calculated from this equation:

Percent inhibition = [1 − (A_s_/A_o_)] × 100
Where A_o_ is the absorbance of the control (containing all reagents except the test compound), and A_s_ is the absorbance of the mixture containing the test compound. The results of all experiments were expressed as mean ± standard deviation.

### 3.10. Antitumor Activity and Cell Toxicity Assay

Antitumor activity and cell toxicity assay of the extracts were determined according to the protocol of Wang et al. [60] with some modifications. Tumor cells, A549, were cultured in Dulbecco’s modified Eagle medium (DMEM) until reaching 80% con-fluence. Trypan blue exclusion method was applied to determine the cell viability. In this experiment, optimum cell viability was above 98% and concentration level was adjusted for further experimentation. Human lymphocyte cells, peripheral blood mononuclear cells (PBMCs), were obtained from healthy volunteers by venipuncture and heparin was used as an anticoagulant. The blood solution was diluted with one-fold sterile phosphate buffer saline (PBS) and was centrifuged with Ficoll-Hypaque gradient centrifugation to separate PBMCs from the other specimens. Briefly, the diluted blood solution was overlaid in Ficoll-Hypaque solution and centrifuged at 1300 rpm, 25 °C for 30 min. The PBMC layer was collected, washed two times with sterile PBS and the redissolved PBMC pellets were treated with RPMI-1640 cell medium with 10% fetal bovine serum (FBS). Cell viability was determined. and the concentration level was adjusted for further experiment.

The 3-(4,5-dimethylthiazol-2-yl)-2,5-diphenyltetrazolium bromide (MTT) assay was used to investigate the cytotoxicity of the three Clauseneae plants extracts on A549 and human lymphocyte cells. Tumor cells (A549) and human lymphocyte cells were cultured in a 96-well tissue culture plate, which contained 5000 and 10,000 cells, in each well, respectively. Different concentrations of the sample solution were added to each well and they were then incubated at 37 °C in a 5% CO_2_ incubator for 24 h. After that, 20 µL of MTT solution (5 mg/mL of MTT in PBS, pH 7.4) was added and the specimens were further incubated at 37 °C for another 4 h. Cell medium was drained out and the formazan dye sediment was dissolved with 100 µL of dimethyl sulfoxide (DMSO). Absorbance was measured at 540 nm and the cell viability ratio was calculated by comparing the absorbance of the wells that did not contain any sample solution.

### 3.11. Ethical Considerations

Concerning the cultured human cells used in this current research, approval to conduct this study was gained from the Ethics Review Committee for Research In-volving Human Research Subjects, by the human ethics committees of the Faculty of Associated Medical Sciences, Chiang Mai University, Thailand (Study Code: AM-SEC-63EM-032).

### 3.12. Statistical Analysis

The results of all experiments were expressed as mean ± standard deviation. Analysis of variance was performed by ANOVA procedure and Duncan’s multiple comparison test was used to determine any significant differences (*p* < 0.05) identified between treatments. The correlation (r) between the two variants was analyzed using the Pearson test. The statistical analyses were performed using SPSS software (SPSS v.25 for windows; IBM Crop., Armonk, NY, USA).

## 4. Conclusions

It can be concluded that matK possesses a good DNA barcode for Clauseneae, especially with regard to the discrimination of *Murraya koenigii* from *Clausena excavata* and *C. harmandiana*. This is due to its ease of amplification and sequence variation. However, a combination with other DNA fragments would increase its degree of identification efficiency. Notably, the extracts obtained from *Clausena excavata*, *C. harmandiana* and *Murraya koenigii* were found to be effective as natural antioxidant, antidiabetic and antihypertension agents and were also found to inhibit human lung cancer cells without any side effects on human normal cells. Moreover, the methanol extract of *M. koenigii* exhibited the strongest degree of biological activities because it possessed the highest amount of containable phenolic compounds and flavonoids. Suggesting that the extracts of the three Clauseneae plants could be promising candidates as natural bioactive agents in various relevant fields, which the biological activities in animal models could be initially studied.

## Figures and Tables

**Table 1:** Chemical composition of *Clausena excavata*, *Clausena harmandiana* and *Murraya koenigii* leaves essential oil.

Rt (min) ^a^	Compound ^b^	% Composition		
		*C. excavata*	*C. harmandiana*	*M. koenigii*
5.617	*α-*Pinene	-	1.05	12.23
5.943	Camphene	-	9.61	0.27
5.998	Sabinene	-	-	0.05
6.685	*β-*Terpinene	-	7.87	-
6.703	*β-*Pinene	-	0.88	0.46
6.952	6-Methyl- 5-hepten-2-one	-	-	0.28
7.068	*β-*Mycene	-	3.69	0.65
7.513	*α-*Phellandrene	-	-	0.63
7.693	3-Carene	0.76	2.41	0.33
8.172	*p*-Cymene	-	1.27	0.53
8.406	D-Limonene	-	7.07	-
8.342	*β-*Phellandrene	-	-	3.67
8.456	Eucalyptol	-	0.5	-
8.616	(E)-Ocimene	-	0.9	-
8.947	*α-*Ocimene	-	0.57	0.78
9.403	*γ-*Terpinene	-	3.57	-
9.705	(Z)*-β-*Terpineol	-	0.4	-
10.419	Terpinolene	0.25	1.38	0.85
10.895	3,7-dimethyl-1,6-Octadien-3-ol	-	-	0.57
11.761	*trans*-1-Methyl-4-(1-methylethyl)-2-cyclohexen-1-ol	-	0.29	-
13.986	4-Terpinenol	-	3.34	0.09
14.849	Estragole	1.23	1.68	0.38
18.805	Anethole	86.72	46.09	26.02
21.137	*α-*Cubebene	-	-	0.15
22.030	Ylangene	-	-	0.16
22.220	Copaene	-	-	0.31
22.400	*cis*-*β-*Guaiene	-	-	0.16
22.925	*β-*Elemene	-	-	0.41
23.067	6,10,11,11-Tetramethyl-tricyclo[6.3.0.1(2,3)]undec-7-ene	-	-	0.23
23.598	*α-*Gurjunene	-	-	0.70
24.202	Caryophyllene	-	0.67	21.15
24.695	Zingiberene	-	-	1.29
24.817	Spathulenol	-	-	1.25
24.978	Eudesma-4(14),11-diene	-	-	0.55
25.434	*α-*Caryophyllene	-	0.17	3.92
26.739	*β-*helmiscapene	-	-	3.81
27.105	*γ-*Elemene	-	0.19	-
27.126	*α-*Selinene	0.34	-	6.10
30.285	Spathulenol	0.56	-	-
30.445	Caryophyllene oxide	-	-	0.85
**Total**		**89** **.** **86**	**93** **.** **60**	**8** **8.** **87**

^a^ Retention time (in minutes). ^b^ Compounds listed in order of elution from a HP-5 MS column. “-”= not detected.

**Table 2:** Total phenolic and total flavonoid contents of methanol extract and essential oil of *Clausena excavata*, *Clausena harmandiana* and *Murraya koenigii*.

Plant Extracts	Percent Yield (%Yield)	Total Phenolic Content (mg GAE/g Extract)	Total Flavonoid Content (mg QE/g Extract)
*C. excavata* methanol extract	5.52 ± 0.32a	22.89 ± 0.93c	30.89 ± 2.15d
*C. excavata* essential oil	0.83 ± 0.06d	9.70 ± 0.72d	23.91 ± 1.98e
*C. harmandiana* methanol extract	4.12 ± 0.19c	19.71 ± 0.83c	39.95 ± 0.63c
*C. harmandiana* essential oil	0.64 ± 0.08d	7.07 ± 0.73d	16.82 ± 1.25f
*M. koenigii* methanol extract	4.92 ± 0.39b	43.50 ± 4.30a	66.13 ± 1.69a
*M. koenigii* essential oil	0.86 ± 0.09d	36.28 ± 1.65b	50.57 ± 1.11b

Average ± standard deviations from three replicates. Different letters in the same column are considered significantly different according to Duncan’s multiple comparison test (*p* < 0.05).

**Table 3:** Antioxidant activity of methanol extract and essential oil of *Clausena excavata*, *Clausena harmandiana* and *Murraya koenigii*.

Plant Extracts	Antioxidant Activity		
	DPPH Free Radical Scavenging (IC_50_, ug/mL)	ABTS Cation Free Radical Scavenging (mg GAE/g extract)	Ferric Reducing Antioxidant Power (mg GAE/g extract)
*C. excavata* methanol extract	904.53 ± 3.23c	88.65 ± 0.71b	16.48 ± 0.72d
*C. excavata* essential oil	2059.29 ± 83.13d	12.27 ± 0.1.75d	5.07 ± 0.17e
*C. harmandiana* methanol extract	2037.66 ± 39.23d	80.11 ± 1.01c	19.07 ± 0.55c
*C. harmandiana* essential oil	2865.26 ± 8.22e	12.83 ± 1.04d	5.37 ± 0.32e
*M. koenigii* methanol extract	95.54 ± 3.46a	118.12 ± 1.01a	48.15 ± 1.21a
*M. koenigii* essential oil	167.74 ± 6.97b	79.52 ± 1.01c	28.22 ± 0.94b

Average ± standard deviations from three replicates. Different letters in the same column are considered significantly different according to Duncan’s multiple comparison test (*p* < 0.05).

**Table 4:** *Alpha-*glucosidase and antihypertensive inhibitory activity of methanol extract and essential oil of *Clausena excavata*, *Clausena harmandiana* and *Murraya koenigii*.

Plant Extracts	*α-*Glucosidase Inhibitory Activity (% Inhibition)	Antihypertensive Inhibitory Activity (% Inhibition)
*C. excavata* methanol extract	49.30 ± 1.10c	47.63 ± 1.11c
*C. excavata* essential oil	36.20 ± 1.49e	38.90 ± 1.05d
*C. harmandiana* methanol extract	41.42 ± 0.65d	58.10 ± 1.75b
*C. harmandiana* essential oil	24.33 ± 0.91f	31.07 ± 1.16e
*M. koenigii* methanol extract	84.55 ± 0.49a	84.95 ± 1.24a
*M. koenigii* essential oil	52.39 ± 1.16b	39.33 ± 1.13d

Average ± standard deviations from three replicates. Different letters in the same column are considered significantly different according to Duncan’s multiple comparison test (*p* < 0.05).

## Data Availability

Data sharing not applicable.
